# Scanning ion conductance microscopy: a convergent high-resolution technology for multi-parametric analysis of living cardiovascular cells

**DOI:** 10.1098/rsif.2010.0597

**Published:** 2011-02-16

**Authors:** Michele Miragoli, Alexey Moshkov, Pavel Novak, Andrew Shevchuk, Viacheslav O. Nikolaev, Ismail El-Hamamsy, Claire M. F. Potter, Peter Wright, S.H. Sheikh Abdul Kadir, Alexander R. Lyon, Jane A. Mitchell, Adrian H. Chester, David Klenerman, Max J. Lab, Yuri E. Korchev, Sian E. Harding, Julia Gorelik

**Affiliations:** 1 Cardiovascular Science, National Heart and Lung Institute, Imperial College London, Dovehouse Street, London SW36LY, UK; 2 Pharmacology and Toxicology, National Heart and Lung Institute, Imperial College London, Dovehouse Street, London SW36LY, UK; 3 Division of Medicine, Imperial College London, Hammersmith Campus, Du Cane Road, London W120NN, UK; 4 Emmy-Noether Group of the DFG, Department of Cardiology and Pneumology, Heart Research Center Göttingen, Georg August University Medical Center, Robert-Koch-Strasse 40, Göttingen 37075, Germany; 5 Heart Valve Research Group, Montreal Heart Institute Research Center, Universite de Montreal, Montreal, Canada; 6 Cardiovascular Science, Heart Science Centre, Harefield Hospital, Imperial College, London, UK; 7 Department of Chemistry, Cambridge University, Lensfield Road, Cambridge CB2 1EW, UK; 8 Faculty of Medicine, MARA Technology University, 40450 Shah Alam, Selangor Darul Ehsan, Malaysia

**Keywords:** scanning ion conductance microscopy, vascular disease, heart failure, electrophysiology, receptors

## Abstract

Cardiovascular diseases are complex pathologies that include alterations of various cell functions at the levels of intact tissue, single cells and subcellular signalling compartments. Conventional techniques to study these processes are extremely divergent and rely on a combination of individual methods, which usually provide spatially and temporally limited information on single parameters of interest. This review describes scanning ion conductance microscopy (SICM) as a novel versatile technique capable of simultaneously reporting various structural and functional parameters at nanometre resolution in living cardiovascular cells at the level of the whole tissue, single cells and at the subcellular level, to investigate the mechanisms of cardiovascular disease. SICM is a multimodal imaging technology that allows concurrent and dynamic analysis of membrane morphology and various functional parameters (cell volume, membrane potentials, cellular contraction, single ion-channel currents and some parameters of intracellular signalling) in intact living cardiovascular cells and tissues with nanometre resolution at different levels of organization (tissue, cellular and subcellular levels). Using this technique, we showed that at the tissue level, cell orientation in the inner and outer aortic arch distinguishes atheroprone and atheroprotected regions. At the cellular level, heart failure leads to a pronounced loss of T-tubules in cardiac myocytes accompanied by a reduction in Z-groove ratio. We also demonstrated the capability of SICM to measure the entire cell volume as an index of cellular hypertrophy. This method can be further combined with fluorescence to simultaneously measure cardiomyocyte contraction and intracellular calcium transients or to map subcellular localization of membrane receptors coupled to cyclic adenosine monophosphate production. The SICM pipette can be used for patch-clamp recordings of membrane potential and single channel currents. In conclusion, SICM provides a highly informative multimodal imaging platform for functional analysis of the mechanisms of cardiovascular diseases, which should facilitate identification of novel therapeutic strategies.

## 1. Introduction

Cardiovascular disease is recognized as the foremost cause of global mortality, and a goal of modern medical research is to uncover the complex mechanisms of this pathology in its natural context. Heart tissue is highly organized in a three-dimensional manner at the levels of the intact tissue (macroscopic level), single cells (microscopic level) and at the nanoscale level of subcellular compartments. Classically, a broad range of conventional techniques has been employed to study these individual levels of organization, while a universal approach integrating this multi-dimensional information has been lacking. For example, biochemical and molecular biological techniques provide insights into various cellular functions but require the destruction of the sample (living tissue or individual cells), which does not allow continuous dynamic measurements. On the other hand, more physiological measurements are possible at the single-cell level but such techniques rarely reach the nanoscale level of the cell organization.

Correlating cardiovascular function at tissue level with cellular functions at single-cell and subcellular levels is crucial for understanding the mechanisms of cardiopathology. However, classical methods do not easily allow this correlation in the same subject of study because they operate at distinct levels of organization. Study of various pathologies requires diverse classical methods. One has to fix and stain tissue and cell preparations for histological and ultrastructural analysis. Cardiac electrophysiology, including arrhythmias, is best studied at the cellular level using intracellular micropipettes to measure action potentials [1] and patch-clamp recordings for transmembrane ion currents [2]. Study of conduction abnormalities requires a tissue or networked *in vitro* models assessed by multiple extracellular electrodes [3,4] or optical recording of impulse propagation [5,6]. For studying cell contraction in normal and pathological hearts, both optical [7] and video methods [8,9] are used. Fluorescence microscopy, e.g. confocal microscopy, allows monitoring of a variety of intracellular signals by fluorescence, such as changes in calcium levels [10], voltage [11] and intracellular energy molecules (ATP, GTP) [12].

In the context of the complex nature of cardiovascular disease, in addition to the use of multiple conventional methods that address individual questions, it would be extremely useful to develop a novel universal technique capable of correlating cell function with morphology, macroscopic structural remodelling in intact tissue, and spatio-temporal aspects of intracellular signalling or ion channel activity measured in single cells and subcellular compartments.

**Figure 1.**
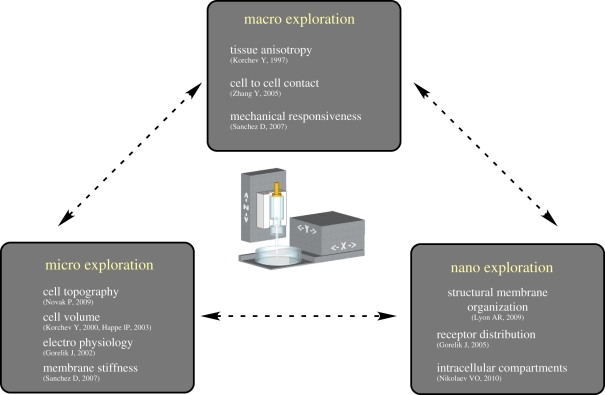
Schematic illustration of scanning ion conductance microscopy as a tool to study tissues and cells at the macroscopic, microscopic and nanoscopic levels of organization. (Online version in colour.)

Scanning ion conductance microscopy (SICM) invented by Hansma *et al*. [13] lately has been developed to image and analyse surface topography of live cells in our group [14–16]. SICM is a non-optical method that uses a nanopipette as a scanning probe to image cell surface structures with nanometre resolution [16]. SICM and a battery of associated innovative methods are unique among current imaging techniques, not only in spatial resolution, but also in the rich combination of imaging modalities with other functional and dynamic methods [10,17,18]. Recently, we have developed a hopping probe ion conductance microscopy (HPICM) [15], using a concept of ‘hopping’ from one imaging point to another, first implemented in SICM as the pulse mode back-step SICM mode [19]. Unlike previous attempts based on this concept, the HPICM managed to obtain nanoscale resolution in highly convoluted live cell samples without compromising the scan speed [15], and has already led to more elaborate techniques for single particle tracking [20] and functional imaging of receptor distribution [18]. The aim of this review is to describe the SICM technique alone or in combination with other optical and electrical methods to perform highly resolved dynamic and integrative analysis of cardiac structure, physiology and mechanisms of cardiovascular disease at the subcellular, cellular and tissue levels (figure 1).

## 2. Principles of scanning ion conductance microscopy

SICM is a non-contact scanning probe microscopy technique which uses a glass nanopipette as a sensitive probe that detects proximity of a surface via a decrease in the ion current flowing through the pipette without any physical contact with the surface. In the conventional implementation of the technique, a continuous feedback mechanism moves the pipette up and down while raster scanning the sample to keep the pipette always in the proximity of the sample surface [13,14,21]. In the recently developed hopping probe HPICM [15], the continuous feedback and raster scanning pattern were abandoned. The nanopipette approaches the surface to measure the height only at selected imaging points and rapidly retracts back to a safe distance before moving laterally onto the next imaging point. This concept, known in scanning electrochemical microscopy as the ‘picking mode’ [22] or ‘force mapping’ in atomic force microscopy [23], was first introduced to SICM by Mann *et al*. [19] as the pulse mode back-step SICM (PMSICM). While successfully extending the applicability of SICM to tall neuronal cell bodies, the PMSICM technique failed to provide nanoscale (≤100 nM) resolution and scanning speeds achieved by the conventional SICM despite an attempt to speed it up by local adjustment of the back-step amplitude in the ‘floating back-step mode’ [24]. Any motion of the probe away from the surface increases the scan speed significantly but this problem was solved for HPICM by using adaptive resolution. Here, a scanning pattern consisting of small squares with a certain number of imaging points is used and the hopping amplitude determined individually and on-the-fly according to the local surface roughness [15]. Combination of all these factors helped the HPICM to achieve a resolution better than 20 nm in highly convoluted cellular samples without compromising the scanning speed [15].

Our described SICM data were recorded from three different set-ups.

Set-up no. 1: ICnano sample scan system (Ionscope Ltd, UK) with 100 × 100 × 100 µm *x*–*y*–*z* piezo-stage for sample movement and 12 µm *z*-axis piezo-actuator for pipette movement. The pipette electrode was connected to the headstage of Multiclamp 700B amplifier (Molecular Devices, USA). Set-up no. 2: custom-modified ICnano sample scan system (Ionscope Ltd, UK) with 100 × 100 µm *x*–*y* piezo-stage for sample positioning and 25 µm *z*-axis piezo-actuator for pipette positioning described in detail previously [15]. The pipette electrode was connected to the CV203BU headstage of the Axopatch 200B patch-clamp amplifier (Molecular Devices). Set-up no. 3: custom-built system with pipette mounted on a three-axis piezo-translation stage (Tritor 100, Piezosystem, Germany) with 80 µm closed-loop travel range in *x*, *y* and *z* directions. The piezo-stage was driven by high-voltage amplifier System ENV 150 (Piezosystem) connected to ICnano scanner controller (Ionscope Ltd). The pipette electrode was connected to Axoclamp 200A (Molecular Devices). Scan heads of all three set-ups were placed on the platforms of Nikon TE200 inverted microscopes (Nikon Corporation, Japan). Pipettes pulled from borosilicate glass with O.D. 1.0 mm and I.D. 0.58 mm (Intracell, UK) using laser puller P-2000 (Sutter Inc.) were used in all experiments.

All three set-ups were operated in the conventional distance-modulated mode [25] or as the HPICM mode as previously described [15] using custom-developed software. When imaging samples of vertical range greater than 12 µm (aortic arch and valve) on set-up no. 1, the *z*-position of the sample was adjusted in synchrony with the *z*-position of the pipette to keep the sample surface at the same distance from the microscope base—a mode developed previously for scanning surface confocal microscopy (SSCM) [26]. Apart from enabling recording of surface fluorescence, the HPICM–SSCM mode effectively extended the *z*-range of set-up no. 1 to 100 µm compared when with just 12 µm in standard HPCIM mode where the *z*-position of the sample is fixed. All other samples scanned with set-up no. 1 as well as the other two set-ups were imaged in standard HPICM. Pipettes with resistance of approximately 100 M*Ω* filled with phosphate-buffered saline were used unless stated otherwise. A 96 × 96 µm topography image of neonatal cardiomyocyte with a pixel width over the cell body of 375 nm took typically 20 min to complete.

## 3. Macroscopic tissue investigation

### 3.1. Cardiac valve and blood vessel cytoarchitecture

Aortic valve disease is a prominent cause of cardiovascular mortality both in the developed and developing world. Surgical valve replacement as the preferred therapeutic option is, in part, owing to the fact that heart valves were thought to passively respond to changes in transvalvular pressures. Recently, it has emerged that heart valves are dynamic structures with a capacity to adapt to their environment [27,28]. This has been borne out by the autograft aortic root replacement (Ross operation), with long-term valve viability and survival comparable to the normal population [29].

**Figure 2.**
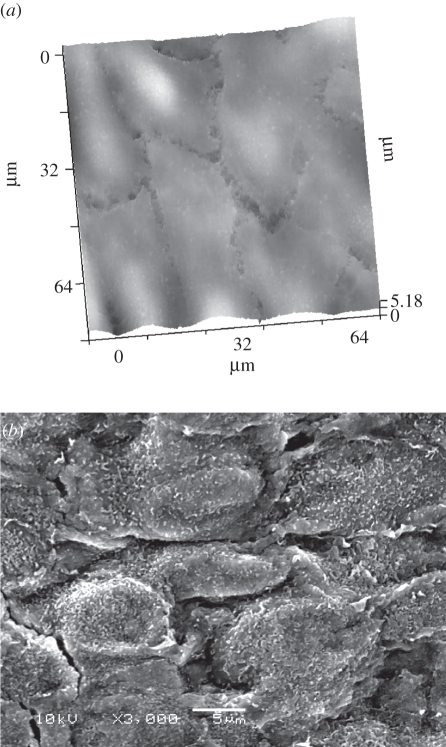
Aortic valve architecture. (*a*) Surface topography of a live explanted porcine aortic valve demonstrating cell shape, size and alignment using scanning ion conductance microscopy (SICM) (A. Moshkov 2010, unpublished data). Effective pixel width 313 nm, scan duration 23 min. Scanning pipette had resistance of 100 M*Ω* and estimated tip diameter of 100 nm. (*b*) Glutaraldehyde-fixed sample of valve imaged using scanning electron microscopy, 2000×, showing cell shape and alignment similar to SICM image in (*a*). Scale bar, 5 µm. (Scanning electron microscope image courtesy of Dr Adrian H. Chester, Cardiovascular Science, Harefield Hospital, Imperial College London, London, UK.)

The aortic valve is composed of a monolayer of endothelial cells lining both sides of the valve, with a mixed population of interstitial cells (smooth muscle cells, fibroblasts and myofibroblasts) lying in between. This is all in a complex haemodynamic and mechanical environment, with endothelial cells from both sides of the valve exposed to different shear stresses [30]. Detailed *in situ* investigations in this topic would be extremely valuable, to understand the pathophysiology and eventual therapies. Figure 2 shows that the SICM can uniquely provide *in situ* evaluation of the topography of aortic valve endothelial cells from the ventricular side of the valve on freshly explanted unfixed aortic valve specimens. The resolution of our SICM-acquired valve topography images was close to that of electron microscopy analysis of the same tissue, which was previously fixed and shaded (figure 2*b*).

**Figure 3.**
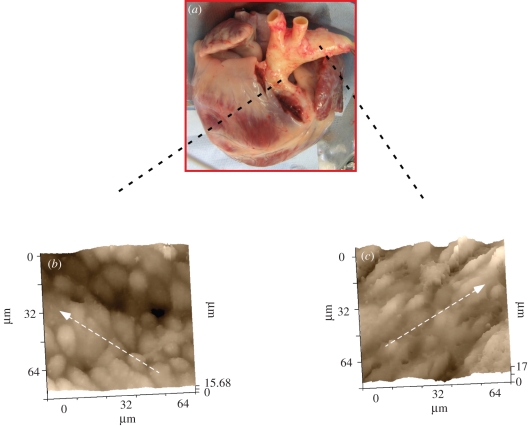
Aorta cell alignment and architecture. (*a*) Intact hearts and attached thoracic aorta of 2-year-old landrace cross pig. (*b*) Representative SICM image of the inner part of the aorta where cells are organized in diffuse pattern. (*c*) SICM image of the outer part of the aorta shows a regularity of cell alignment, which indicates that this area is exposed to higher stress. Dashed arrows indicate blood flow direction. Effective pixel width in both images 625 nm, scan duration 13 min. Scanning pipette had a resistance of 100 M*Ω* and an estimated tip diameter of 100 nm. (A. Moshkov 2010, unpublished data.) (Online version in colour.)

Atherosclerosis is an even more important cause of death worldwide, with vessel inflammation, endothelial dysfunction and plaque formation in arterial walls. Plaques predominantly occur inside abrupt changes in vessels (branch points, bifurcations and the inner curvature of vessels) and the geometric specificity is probably owing to variation in shear stress as a function of flow velocity and viscosity [31,32] with plaques accumulating at atheroprone regions of low or oscillatory shear. Regions of high laminar shear stress are atheroprotected. The mechanism of atheroprotection by shear stress is yet to be fully determined. A difference in morphology has been identified using SICM. Owing to prominent undulations of the tissue surface, we narrowed the scan to an 80 × 80 µm region in SSCM mode [33]. Interestingly, using this approach we found differences in morphology between cells in the atheroprone (figure 3*b*, inner aortic arch) and the atheroprotected (figure 3*c*, outer aortic arch) regions of intact aorta. The inner arch cells were disordered with a cobblestone appearance, whereas the outer arch cells were elongated and aligned in the direction of blood flow, in accordance with findings using atomic force microscopy [34] and scanning electron microscopy [35].

## 4. Microscopic investigation

### 4.1. Topographical changes in failing cardiomyocytes

**Figure 4.**
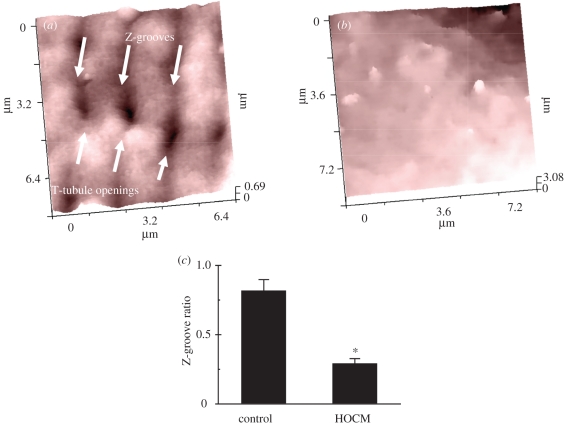
(*a*) Typical surface topography image of a healthy adult cardiomyocyte. Well-organized striation and Z-grooves can be observed. Effective pixel width 125 nm, scan duration 4 min. (*b*) Surface topography image of an adult cardiomyocyte from HOCM patients shows an absence of T-tubules in this 9 × 9 µm area of the cell. (*c*) Z-grooves ratio index quantification demonstrates a significant difference in HOCM compared with control cells (*n* = 5 ± s.e. in both control and HOCM patients, *p* < 0.05 Student's *t*-test). Scanning pipette had a resistance of 100 M*Ω* and an estimated tip diameter of 100 nm. Modified from Lyon *et al*. [37] with permission. (Online version in colour.)

Structural remodelling of the heart, which can lead to heart failure (HF) and cardiac arrhythmias [36], ranges from three-dimensional reorganization to redistribution of the ion channel repertoire and receptors on the cell surface. This is manifest at the tissue level typically involving structural disorganization and hypertrophy of cardiomyocytes. SICM has the capability to resolve this in live cardiomyocytes, with hypertrophic obstructive cardiomyopathy (HOCM) and dilated cardiomyopathy cardiomyocytes showing drastically reduced Z-grooves organization, which lead to the further functional abnormalities [37]. Figure 4 describes the surface characteristics of an adult human cardiomyocyte with the surface structures resolved with SICM. Recently, we introduced a new parameter that describes the integrity of the cardiomyocyte surface called the Z-groove index [21]. SICM images clearly show the surface topography of the cardiomyocyte (figure 4*a*). The domed crest between the Z-grooves, as well as the T-tubule openings are very clear in rat myocytes. Profile measurements showed that the spacing between Z-grooves was approximately 2 µm, corresponding to the predicted sarcomere length for quiescent ventricular myocytes. We showed that different pathological conditions in cardiomyocytes from rats and humans change this index. For example, in cardiomyocytes derived from dilated cardiomyopathy patients, the Z-groove index is reduced, compared with healthy cells [37]. Here, we further investigated surface structures of healthy and diseased cardiomyocytes. Cardiomyocytes from patients with HOCM contained fewer Z-grooves and therefore their Z-groove index was lower than in normal cells. Figure 4*a* shows a control human cardiomyocyte with striated pattern on the surface with T-tubule openings distributed at regular intervals. Z-grooves are pronounced, and the Z-groove index is 0.86 (figure 4*c*). In sharp contrast, cardiomyocytes from a patient with HOCM show dramatic changes in surface structure, with flattening and loss of Z-groove definition (figure 4*b*). The Z-groove index in HOCM cells was as low as 0.15.

### 4.2. Volume measurement in cellular hypertrophy

Regulation of cell volume is fundamental to cellular homeostatic mechanism. Changes in cardiomyocyte structure are frequently accompanied by changes in cell volume [38]. To investigate the mechanisms associated with cell volume regulation, it is important to use an appropriate technique which is capable of precisely measuring cell volume while maintaining the cell integrity. The most commonly used techniques are based on continuous monitoring of loaded cell reagent (ions or fluorescence dyes), using quantitative fluorescent microscopy [39,40] or ion-sensitive microelectrodes [41]. In yet another experimental system, the relative changes in cell volume can be assessed by a simple electrophysiological method for continuous height-measurement [42]. One of the most advanced techniques uses scanning laser confocal microscopy but even this method is limited by photodynamic damage and special requirements are needed for specimen preparation [43]. A direct cardiomyocyte hypertrophy is frequently assessed using two conventional approaches: the first calculates using a circular formula, the cross-sectional area (circular cell profile, *πr*^2^) per cell length [44]. The second uses a ‘coulter cell counter’ associated with software based on a predetermined cell shape factor [45].

**Figure 5.**
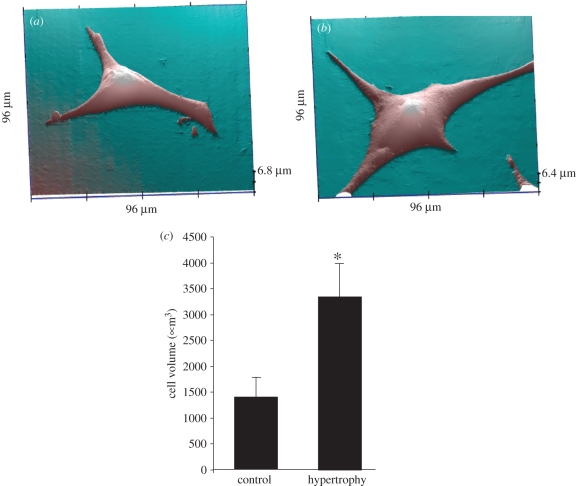
Neonatal rat ventricular myocytes were exposed to PE for 48 h to induce hypertrophy. (*a*) The 96 × 96 µm scan of cardiomyocyte after 48 h in culture under control conditions. The process on the right side of the cardiomyocyte appears to be cropped only owing to the angle of view. (*b*) Same size scan performed on a different cardiomyocyte exposed to 10 µmol l^−1^ PE. (*c*) Average cell volume in control and hypertrophic cardiomyocytes (*n* = 15 ± s.d., *p* < 0.05 Student's *t*-test). Asterisk denotes significant difference compared with control. Scanning pipette had a resistance of 100 M*Ω* and an estimated tip diameter of 100 nm. Effective pixel width in (*a*,*b*) is 375 nm over the cell body and 750 nm over the empty area. (M. Miragoli & P. Novak 2010, unpublished data.) (Online version in colour.)

SICM, to our knowledge, is the most appropriate technique for studying cell hypertrophy directly *in vitro*, without damaging the sample [46,47]. Hopping probe SICM is a fairly simple modification and an accurate method to measure cardiac hypertrophy (figure 5). Although the large surface area (approx. 100 × 100 µm) of a typical hypertrophic cardiomyocyte limits the resolution of the image when recorded with the current implementation of HPICM to just 400–200 nm, it still allows an accurate cell volume calculation. One-day old neonatal rat ventricular cardiomyocytes, originating from 12 rats, were grown on 22 mm coverslips. After 24 h, six coverslips were kept as ‘control’ and the other six exposed to 10 µmol l^−1^ phenylephrine (PE). The volume of randomly selected 15 cells in both groups was analysed using the topography data recorded by HPICM. As expected, neonatal rat ventricular cardiomyocytes treated with PE showed a significant increase in volume. Figure 5*a* presents a control cardiomyocyte cultured for 48 h without PE. The average volume in the control group was 1388 ± 384 µm^3^. Culturing for 48 h in PE medium increased the total cellular volume to 3389 ± 599 µm^3^ (figure 5*b*,*c*). The volume of cardiomyocytes cultured in the PE medium was underestimated in few cases (three cells out of 15) owing to cell processes exceeding the scan area (figure 5*b*). Based on the volume of other processes included in the area, the resulting error was estimated to be no more than 4 per cent, five times less then the standard deviation of the mean volume in control (approx. 3.5% of the mean, figure 5*c*). The 132 per cent volume increase in cardiomyocytes cultured in the PE medium remained highly significant (*p* < 0.01) with as well as without the three cells affected by the volume underestimation.

### 4.3. Cardiac contractility

Cardiac contraction has been classically studied at the organ level, with parameters such as ejection fraction, pressure–volume loops and the Frank–Starling curve being of the most importance. At the cellular level, it translates to the study of sarcomere length and force–velocity relationship. Moreover, cardiac contractility is intimately regulated by multiple humoural activities (e.g. circulating catecholamines), which work in concert and modulate the normal function of the organ. In HF, the parameters of cardiac contractility are extremely important as clinical indexes, for example, reduction of inotropy may lead to a fall in stroke volume, thereby decreasing ejection fraction.

For investigating contractility *ex vivo*, a classical approach which is still in use today calls for the direct measurement of myofibril contraction using a cantilever force probe attached to a glass needle mounted on a lever arm of a length control monitor [48]. Another popular technique uses live video-imaging of sarcomere shortening and other parameters in isolated adult cardiomyocytes (e.g. IonOptix system) [49]. Recently, Dr Parker's group described a new interesting motion measurement technique while culturing cardiomyocytes on a patterned surface to provide geometrically defined areas of growth [50].

Here, we show that SICM is a suitable technique for the (i) identification of contractile cell phenotype, i.e. a cluster of human embryonic stem cell-derived cardiomyocytes (hESCMs) among other cells that derived from stem cells and (ii) simultaneous investigation of inotropy and Ca^2+^ transient in neonatal ventricular myocytes, in combination with optical recording using a fast video camera. For both types of measurement, the preparations are mounted on a 0.1 mm thick glass coverslip. In these attached cells, the shortening of the myocyte is constrained by the attachment points, so the vertical displacement of the pipette as the cell thickens with each beat is a useful surrogate.

**Figure 6.**
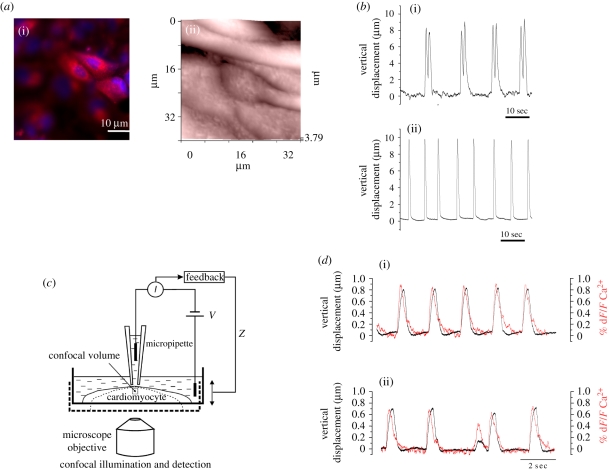
Measurement of contraction by SICM in cluster of (i) human embryonic stem cell-derived cardiomyocytes (hESCMs) and (ii) neonatal rat ventricular myocytes. (*a*) (i) hESCMs stain with myosin heavy chain. (ii) Topographical 32 × 32 µm image of cluster of hESCM using SICM. (*b*) (i) Contraction of hESCM cluster in the presence of (i) doxorubicin and esmolol (ii) resulting in changes in pipette vertical displacement of SICM. As expected, the presence of doxorubicin affects cardiac contraction; this condition is restored by esmolol (ii). (*c*) Technical scheme of SICM/Ca^2+^ dynamics for concurrent measurement of contraction and intracellular Ca^2+^ transient. Simultaneously, the light emission of the stained cell loaded with Fluo-4 AM was detected by a custom-made photomultiplier tube apparatus. (*d*) Overlapped traces of Ca^2+^ transient (normalized at % d*F*/*F*) and contraction (vertical displacement). (i) Control cluster of cardiomyocytes denote spontaneous firing (approx. 60 b.p.m.). (ii) Same as (i) but in the presence of taurocholic acid that affects calcium transient amplitude and contraction (*p* < 0.05, Student's *t*-test). Scanning pipette had a resistance of 100 M*Ω* and an estimated tip diameter of 100 nm. Topography image in (*a*) was recorded in the conventional distance-modulated mode with pixel number set to 1024 × 256. Scan duration was 23 min. Modified from Gorelik *et al*. [51,52] with permission. (Online version in colour.)

In SICM, the electrical feedback system keeps the distance between the tip of the pipette and the cell surface constant, thereby providing information about the movement of the cell surface if it moves, as in contracting cells (figure 6*c*). The vertical displacement of the pipette can be recorded and analysed. Using SICM, we found that in hESCMs clusters only a small fraction of cells are actually contracting, a characteristic of differentiated cardiomyocytes [53] (figure 6*a*(ii)). Histochemically, those differentiated cells express cardiomyocytes markers, such as myosin heavy chain (figure 6*a*(i)). Application of drugs known to perturb contraction is useful to evaluate the state of differentiation of hESCM clusters. The most differentiated cells react to the arrhythmogenic action of doxorubicin and are protected from this action by esmolol (figure 6*b*) [51].

Another example illustrates the use of SICM with neonatal rat ventricular myocyte, an interesting model for studying arrhythmia *in vitro* [10]. Normally, these cardiomyocytes in culture beat constantly at approximately 60 b.p.m. (figure 6*d*(i)). In the presence of taurocholic acid, known to affect Ca^2+^ homeostasis in neonatal cardiac cells, the cells start to show signs of arrhythmia and desynchronized beating, and Ca^2+^ amplitude is also reduced (figure 6*d*(ii)) [52].

### 4.4. Cardiac electrophysiology

**Figure 7.**
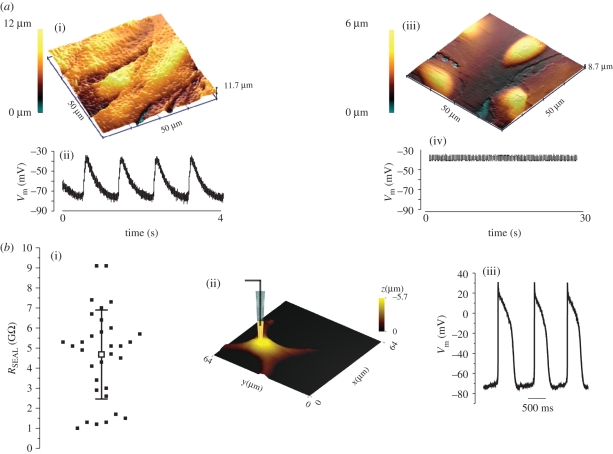
(*a*) Illustration of the use of SICM for electrophysiological measurement. (i) A scan of a region of neonatal rat ventricular myocytes monolayer with highest thickness = 12 µm. (ii) Representative resting *V*_m_ measured with SICM (*n* = 20). (iii) Scan of a monolayer of cardiac myofibroblasts. Note that the highest thickness (6 µm) corresponds to the region above the nuclei. (iv) SICM allowed successful measurement of *V*_m_ in the region of the cell surface above the nucleus (*n* = 20). Pipette had a resistance of approximately 20 M*Ω* and an estimated tip diameter of approximately 500 nm. Effective pixel width in topography images was 400 nm, scan duration 20 min. (*b*) Whole-cell recording in neonatal rat ventricular myocytes using SICM. (i) Resistance of the pipette used for whole-cell recording. The distribution of the seal resistance *R*_SEAL_ measured after obtaining stable gigaseal configuration (solid squares). (ii) Schematic of a patch-pipette performing whole-cell recording in a neonatal rat ventricular myocyte. (iii) Example of a whole-cell action potential recording in a neonatal rat ventricular myocytes in the current-clamp mode showing spontaneous action potential firing. Values were corrected for liquid junction potential (*n* = 42). Pipettes used for whole-cell patch-clamp recording had resistance in the range of 6–9 M*Ω* and estimated diameter of 1.7–1.1 µm diameter. (M. Miragoli 2009 & P. Novak 2010, unpublished data.) (Online version in colour.)

The use of a glass pipette containing an electrode connected to an amplifier immediately calls for the application of other commonly used electrophysiological techniques such as patch-clamp and intracellular voltage measurements. The SICM is perfectly suited for both techniques and it further improves their performance. Two main reasons place SICM as an ideal instrument for intracellular measurement: (i) precise determination of the cell morphology before impalement and (ii) nanometric, automatic and vertical approach. The SICM permits the selection of the location on the cell surface by a well-controlled vertical approach with nanometre precision, resulting in the easy formation of a contact gigaseal with the membrane bilayer. Figure 7*a*(i) (scan time 7 min) shows a 50 × 50 µm topographical scan of neonatal rat ventricular myocytes with a tallest peak of 12 µm. Using a pipette filled with 3 mol l^−1^ KCl and the precise three-dimensional position control of the SICM, we could place the pipette at any place on the cell surface. When lowering the pipette, the access resistance started to increase, indicating a ‘quasi-attachment’ onto the cell surface. A small additional, automatic downward advancement of 50–100 nm resulted in the pipette tip penetrating the membrane. After 1 min of stabilized impalement, *V*_m_ was recorded (figure 7*a*). In this example, resting *V*_m_, as expected, was −79 mV and the cardiac monolayer showed spontaneous electrical activity with depolarizing transients.

The impalement of cardiomyocytes is usually uncomplicated owing to the rod-like shape of these cells; this is not the case for much flatter cells such as myofibroblasts. Cultured myofibroblasts are extremely flat and only the regions above the nuclei represent a suitable location for impalement; therefore, these areas can be accurately selected. Furthermore, when these cells merge into a network, it becomes difficult to distinguish the nuclei of individual cells. Cardiac myofibroblasts are much thinner cells than cardiomyocytes and therefore the impalement is not as straightforward. The highest peak and thus the most successful impalement were possible only in the regions above the nuclei (figure 7*a*(iii)). A pre-scan of a myofibroblast monolayer using the same pipette, which was later used for electrophysiological recording, indicated that the regions above the nuclei were only 6 µm high. In this case, the vertical approach needs to be more accurate, and we reduced the vertical downward steps to only 10–20 nm. Figure 7*a* shows that this monolayer produced a resting membrane potential of −39 mV and did not show spontaneous electrical activity, consistent with the nature of these cells [54].

A further improvement is the vertical approach with nanoscale precision that, together with the electrical feedback, keeps the pipette nanometers above the selected location. This facilitates a clean and stable cell-attach lasting several minutes. With action potential recording in whole-cell configuration, we attained gigaseals in 85.7 per cent of cells (approx. 4.5 G*Ω*, figure 7*b*).

**Figure 8.**
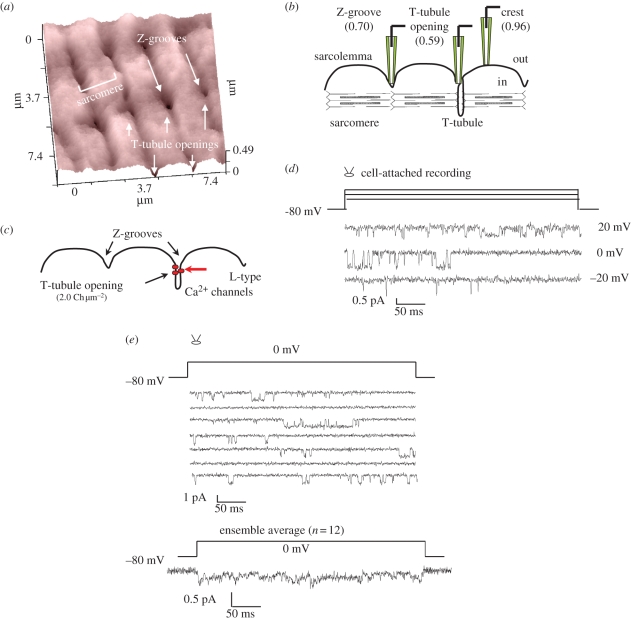
L-type Ca^2+^ channel distribution in the cardiac myocytes sarcolemma: mapping of ion channels by the high-resolution scanning patch-clamp technique. (*a*) Experimental topographic image of a representative rat cardiomyocyte sarcolemma. Z-grooves, T-tubule opening and characteristic sarcomere units are marked. (*b*) Functional schematic of sarcomere units showing the position of the probed region (Z-groove, T-tubule opening and scallop crest). Probabilities of forming a gigaseal as a function of surface position shown in parentheses. (*c*) Statistical distribution of L-type Ca^2+^ channels with the highest density near the T-tubule opening. (*d*) Cell-attached Ba^2+^ current transients at voltages of +20, ±0, −20 mV. (*e*) Several current transients elicited at 0 mV from one patch and ensemble average of 12 transients showing typical L-type inactivation kinetics. Scanning pipette had a resistance of 100 M*Ω* and an estimated tip diameter of approximately 100 nm. Topography was recorded in the conventional distance modulated mode with pixel number set to 1024 × 256. Scan duration was 20 min. Modified from Gu *et al*. [55], with permission. (Online version in colour.)

Obtaining a topographical image before an intervention permits the accurate selection of the location for the cell-attach (figure 8). SICM combined with patch-clamp technique formed a unique ‘smart’ patch-clamp system [17,56] on the surface of adult cardiomyocytes, where ion channels are confined in determined regions (figure 8*b*,*c*). We demonstrate a measurement of Ca^2+^ L-type channels within T-tubules system by measuring Ba^2+^ current transient at voltage of +20, 0 and −20 mV (figure 8*d*) in cell-attach configuration and a typical L-type inactivation kinetics (figure 8*e*).

## 5. Nanoscopic investigation

### 5.1. Receptor localization

In addition to the measurements of electrical properties in various subcellular regions with distinct structure, we studied how the SICM technique can increase the resolution of conventional microscopy in correlating subcellular signalling responses with the membrane topography.

**Figure 9.**
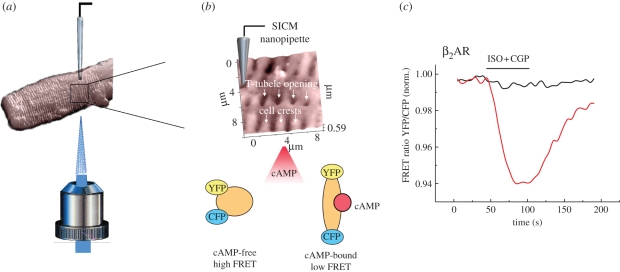
Principle of the SICM/FRET technique and its use to study βAR localization in cardiomyocytes. (*a*) SICM image (32 × 32 µm) of an adult rat cardiomyocyte acquired using a nanopipette from the top of the cell. The sample is positioned on an inverted epifluorescent microscope, so that recordings of cellular fluorescence can be performed. (*b*) Inset shows a 10 × 10 µm scan of the cardiomyocyte surface with characteristic structural features (cell crests, Z-lines and T-tubule openings). Effective pixel width was 156 nm, scan duration 4 min. The cells are expressing a FRET-based cAMP sensor Epac2-camps, which reports changes in intracellular cAMP levels after local cell surface stimulation via an SICM nanopipette with β1AR or β2AR selective ligands applied either into a T-tubule opening or onto the cell crest. Binding of cAMP to the sensor causes a change in its conformations, which results in a longer distance between the fluorophores (CFP and YFP) and lower FRET signal. (*c*) Stimulation of β1ARs in both T-tubular (red line) and cell crest region (black line) results in a decrease of FRET, which reflects an increase in cAMP levels. In contrast, β2AR induces cAMP signals only when stimulated in the T-tubule, but not on the cell crest (*n* = 9). Scanning pipette had a resistance of 100 M*Ω* and an estimated tip diameter of 100 nm. Modified from Nikolaev *et al*. [18] with permission. (Online version in colour.)

G-protein-coupled receptors such as β-adrenergic receptors (βARs) and M_2_ muscarinic receptors play a central role in regulating cardiac function and disease. We recently developed a novel functional approach that combines SICM with local ligand application and fluorescence resonance energy transfer (FRET)-based measurements of cAMP production by locally activated receptors (figure 9*b*) [18]. Using this hybrid SICM/FRET technique, we showed that β_2_AR are selectively localized in the T-tubules of healthy adult rat cardiomyocytes (figure 9*b*,*c*), while β_1_AR are evenly distributed across the cell membrane. Importantly, cells isolated from rats after myocardial infarction revealed a redistribution of β_2_AR, which now appeared in non-tubular areas of detubulated failing cardiomyocytes [18]. Redistribution of this receptor also resulted in changes of subcellular compartmentation of cAMP signals, which might play an important role in the development of cardiac disease. Figure 9 shows that one can combine SICM with FRET to analyse the precise distribution of various membrane receptors with a few-hundred nanometre resolution and to correlate disease-driven changes in cell surface morphology with alterations in intracellular signalling. We believe that this approach provides another multi-parametric possibility to study functionally relevant signalling compartments in cardiac cells and to investigate how receptor distribution and the subcellular mechanisms of receptor-mediated downstream signalling are changed in cardiac disease.

## 6. Conclusion and perspectives

A hierarchical level of organization within living cardiovascular tissues requires the application of various methods to study the structure and function. High-resolution multi-parametric techniques and investigation of cell function at various levels of tissue organization (from macroscopic to nanoscopic) is one of the major current interests in cardiovascular biology. The aim is to correlate physiological function of the tissue with cellular and subcellular processes. However, contemporary technologies diverge and they are difficult to use together on the same biological substrate. SICM represents a versatile universal platform for all these studies. Using various modification of the SICM technique, one can investigate such diverse processes as arrhythmias, HF, atherosclerosis, hypertrophy, valvular heart disease and mechanical dysfunction of the heart. Studies using SICM may facilitate efforts to uncover the mechanisms of various cardiovascular diseases and to identify potential novel therapeutic strategies.
